# Association of injury after prescription opioid initiation with risk for opioid-related adverse events among older Medicare beneficiaries in the United States: A nested case-control study

**DOI:** 10.1371/journal.pmed.1004101

**Published:** 2022-09-22

**Authors:** Yu-Jung Jenny Wei, Cheng Chen, Ting-Yuan David Cheng, Siegfried O. Schmidt, Roger B. Fillingim, Almut G. Winterstein

**Affiliations:** 1 Department of Pharmaceutical Outcomes and Policy, College of Pharmacy, University of Florida, Gainesville, Florida, United States of America; 2 Center for Drug Evaluation and Safety, University of Florida, Gainesville, Florida, United States of America; 3 Division of Outcomes and Translational Sciences, College of Pharmacy, The Ohio State University, Columbus, Ohio, United States of America; 4 Department of Biostatistics, Epidemiology and Informatics, University of Pennsylvania, Philadelphia, Pennsylvania, United States of America; 5 Division of Cancer Prevention and Control, Department of Internal Medicine, College of Medicine, The Ohio State University, Columbus, Ohio, United States of America; 6 Department of Community Health and Family Medicine, College of Medicine, University of Florida, Gainesville, Florida, United States of America; 7 Pain Research and Intervention Center of Excellence, University of Florida, Gainesville, Florida, United States of America; 8 Department of Epidemiology, Colleges of Medicine and Public Health and Health Professions, University of Florida, Gainesville, Florida, United States of America; Universite de Paris Faculte de Sante, FRANCE

## Abstract

**Background:**

Injury, prevalent and potentially associated with prescription opioid use among older adults, has been implicated as a warning sign of serious opioid-related adverse events (ORAEs) including opioid misuse, dependence, and poisoning, but this association has not been empirically tested. The study aims to examine the association between incident injury after prescription opioid initiation and subsequent risk of ORAEs and to assess whether the association differs by recency of injury among older patients.

**Methods and findings:**

This nested case-control study was conducted within a cohort of 126,752 individuals aged 65 years or older selected from a 5% sample of Medicare beneficiaries in the United States between 2011 and 2018. Cohort participants were newly prescribed opioid users with chronic noncancer pain who had no injury or ORAEs in the year before opioid initiation, had 30 days or more of observation, and had at least 1 additional opioid prescription dispensed during follow-up. We identified ORAE cases as patients who had an inpatient or outpatient encounter with diagnosis codes for opioid misuse, dependence, or poisoning. During a mean follow-up of 1.8 years, we identified 2,734 patients who were newly diagnosed with ORAEs and 10,936 controls matched on the year of cohort entry date and a disease risk score (DRS), a summary score derived from the probability of an ORAE outcome based on covariates measured prior to cohort entry and in the absence of injury. Multivariate conditional logistic regression was used to estimate ORAE risk associated with any and recency of injury, defined based on the primary diagnosis code of inpatient and outpatient encounters. Among the cases and controls, 68.0% (*n* = 1,859 for cases and *n* = 7,436 for controls) were women and the mean (SD) age was 74.5 (6.9) years. Overall, 54.0% (*n* = 1,475) of cases and 46.0% (*n* = 1,259) of controls experienced incident injury after opioid initiation. Patients with (versus without) injury after opioid therapy had higher risk of ORAEs after adjustment for time-varying confounders, including diagnosis of tobacco or alcohol use disorder, drug use disorder, chronic pain diagnosis, mental health disorder, pain-related comorbidities, frailty index, emergency department visit, skilled nursing facility stay, anticonvulsant use, and patterns of prescription opioid use (adjusted odds ratio [aOR] = 1.4; 95% confidence interval (CI) 1.2 to 1.5; *P* < 0.001). Increased risk of ORAEs was associated with current (≤30 days) injury (aOR = 2.8; 95% CI 2.3 to 3.4; *P* < 0.001), whereas risk of ORAEs was not significantly associated with recent (31 to 90 days; aOR = 0.93; 95% CI 0.73 to 1.17; *P* = 0.48), past (91 to 180 days; aOR = 1.08; 95% CI 0.88 to 1.33; *P* = 0.51), and remote (181 to 365 days; aOR = 0.88; 95% CI 0.73 to 1.1; *P* = 0.18) injury preceding the incident diagnosis of ORAE or matched date. Patients with injury and prescription opioid use versus those with neither in the month before the ORAE or matched date were at greater risk of ORAEs (aOR = 5.0; 95% CI 4.1 to 6.1; *P* < 0.001). Major limitations are that the study findings can only be generalized to older Medicare fee-for-service beneficiaries and that unknown or unmeasured confounders have the potential to bias the observed association toward or away from the null.

**Conclusions:**

In this study, we observed that incident diagnosis of injury following opioid initiation was associated with subsequent increased risk of ORAEs, and the risk was only significant among patients with injury in the month before the index date. Regular monitoring for injury may help identify older opioid users at high risk for ORAEs.

## Abstract 2

In a nested case-control study, Yu-Jung Jenny Wei and colleagues study associations between injury following prescription opioid initiation and risk of an opioid-related adverse event among older Medicare beneficiaries in the US.

## Author summary

**Why was this study done?:**

- Injury has been hypothesized as a warning sign of serious opioid-related adverse events (ORAEs) including opioid misuse, dependence, and poisoning, which has increased over the past decade among older adults.
- Yet, the hypothesis that injury after opioid therapy among older adults may act as an important warning sign of ORAEs remains untested.

**What did the researchers do and find?:**

- The primary analysis assessed the occurrence of incident injury at any time after prescription opioid initiation and before an incident ORAE for 2,734 cases and over the same period for their 10,936 matched controls who did not experience an ORAE, selected from a cohort of Medicare older patients diagnosed with chronic noncancer pain who were new users of prescription opioids. The secondary analysis assessed whether recency of injury was associated with risk for ORAEs.
- We found that patients with (versus without) injury after opioid therapy had higher risk of ORAEs after adjustment for time-varying confounders.
- We also found that increased risk of ORAEs was associated with current (≤30 days) injury, whereas risk was not significantly associated with recent (31 to 90 days), past (91 to 180 days), and remote (181 to 365 days) injury preceding the incident diagnosis of ORAE for cases or equivalent matched date for controls.

**What do these findings mean?:**

- An incident diagnosis of injury following opioid initiation was associated with subsequent increased risk of ORAEs, and the risk was only significant among patients with injury in the month before the diagnosis of ORAE or matched date.
- Regular monitoring for injury may help identify older opioid users at high risk for ORAEs.

## Introduction

Older adults in the United States have experienced substantial increases in opioid-related adverse events (ORAEs), including opioid use disorder (OUD), opioid overdose (OD), and opioid misuse in the last 15 years despite a decrease in opioid prescribing. While the overall rates of ORAE are relatively low in older versus younger populations, between 2006 and 2016, adults aged 65 years or older had the largest increase of all age groups in incident diagnosis of OUD or OD (14.2-fold versus 3.5-fold for adults 18 to 64 years of age) [1]. This finding parallels a federal study showing that hospitalizations and emergency department (ED) visits associated with OUD and OD among older adults increased by 34.3% (from 199.3 to 267.6 per 100,000 population) and 74.2% (from 44.7 to 77.9 per 100,000 population), respectively, from 2010 to 2015 [2]. Opioid misuse, defined as the use of opioids without a prescription or for reasons or in ways other than as prescribed, doubled from 484,000 persons (1.1%) in 2002 to 880,000 persons (2.0%) in 2014 among older adults [3]. The majority (98%) of opioid misuse episodes among older adults involves prescription opioids, with only 2% involving non-prescribed opioids (i.e., diverted or illicit sources) [4]. The increase in OUD, OD, and opioid misuse contrasts with the decrease in opioid prescribing [5] and signals an urgent need to identify factors beyond opioid prescribing that may contribute to ORAEs to inform interventions for older populations [6].

Injury is prevalent and potentially associated with prescription opioid use among older adults and has been postulated to be an early warning sign of ORAEs. Injury is considered an event that potentially reflects opioid misuse and user disorder and emerges between opioid initiation and opioid-related harms [7]. This presumption is supported by a 217% increase in the population-adjusted rate of ED visits with a diagnosis of opioid misuse among older adults (from 37.8 per 100,000 in 2006 to 119.9 per 100,000 in 2014), with injuries being more prevalent among older adults with an opioid misuse-related ED visit compared with the older population in general (30.3% versus 20.0%, *P* < 0.001) [8]. Furthermore, patients with (versus without) injuries were 1.4 times as likely to report persistent opioid use [9], which increases the risk for opioid dependence and overdose [10,11].

To date, the hypothesis that injury after opioid therapy among older adults may act as an important warning sign of ORAEs remains untested. Thus, this study aimed (1) to examine whether injury after initial use of prescription opioids was associated with subsequent risk of an ORAE; and (2) to assess whether the association differed by the recency of injury preceding an ORAE among US older adults. This second aim allowed for understanding whether the timing of injury matters in estimating future risk of ORAEs among older adults.

## Methods

### Study design and data sources

We conducted a nested case-control study in a cohort of beneficiaries with chronic noncancer pain who were at least 65 years of age and had initiated prescription opioids, assembled from a 5% random Medicare sample from January 1, 2011 to December 31, 2018. Medicare is a federal health insurance program for Americans who are 65 or older, those under 65 who have a disability, or those with end-stage renal disease. In 2018, a total of 61.5 million Americans were enrolled in Medicare, with 86% of them due to age [12,13]. Medicare provides coverage for Part A (inpatient service), Part B (office-based visits), and Part D (prescription drugs) for its fee-for-service beneficiaries [12]. In this study, we used a 5% sample of Medicare beneficiaries provided by the Centers for Medicare and Medicare Services (CMS), and the 5% sample is randomly selected based on the last 2 digits of the beneficiary’s claim account number according to standard CMS processes [14]. Medicare claims data have been widely used for observational studies, with good agreement in various disease conditions (e.g., 89% for hip fracture) [15], procedures (e.g., 92.2% for pacemaker implantation) [16], and prescription drugs (between 95% and 99%) [17], in comparison with medical records.

**Fig 1:**
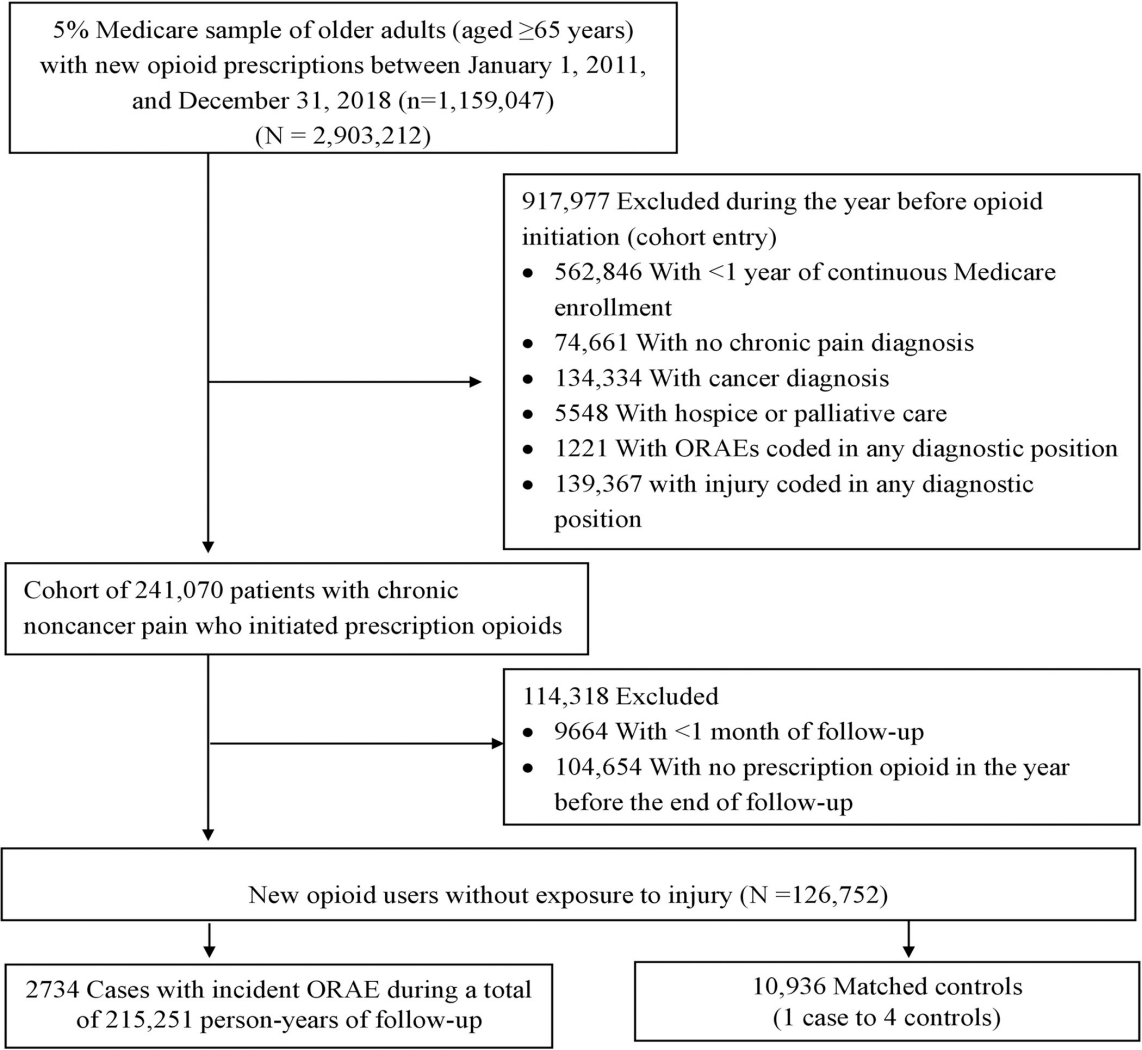
Cohort inclusion flowchart for the nested case-control study sample. ORAE, opioid-related advance event.

As depicted in **Fig 1**, a nested case-control design allowed for (1) including all identified ORAE cases, which are relatively rare among older adults compared with younger populations [1]; and (2) studying the association between recency of injury exposure and risk for ORAEs by flexibly modeling the exposure at varying proximities to the event date [18]. The study was approved by the University of Florida Institutional Review Board and followed the Strengthening the Reporting of Observational Studies in Epidemiology (STROBE) reporting guideline (**S1 STROBE Checklist)**. Data analyses were performed as per a prespecified protocol between January and December 2021 (**S1 Text**).

### Identification of the study cohort

We identified individuals 65 years of age or older who were naïve to prescription opioids defined as having no opioid prescription fill within 1 year before the date of their first dispensed opioid prescription (excluding buprenorphine sublingual tablets and buprenorphine-naloxone combinations indicated for treatment of OUD or OD; **S1 Table)**, which was set as the cohort entry. During the 1-year pre-cohort entry, patients were required to have the following: (1) a primary or secondary diagnosis of a chronic pain condition (**S2 Table**) to ensure a relatively homogeneous cohort regarding pain conditions; (2) continuous enrollment in Medicare Parts A (inpatient), B (outpatient provider), and D (prescription drug) without insurance coverage from health maintenance organizations or employer-sponsored plans; and (3) no cancer diagnosis, hospice care, or palliative care because of the different pain management and treatment goals considered for these patients. We excluded patients who had received a diagnosis of an injury of interest (defined in the exposure measurement) or ORAE outcome (defined in the outcome measurement) in any diagnostic position during the year before cohort entry. We then followed eligible patients until the earliest date of ORAE outcome, a cancer diagnosis, receipt of palliative or hospice care, death, Medicare disenrollment, or study end (December 31, 2018). We further restricted the study cohort to individuals who had 30 or more days of follow-up, a minimum time window required to detect potential injury after prescription opioid initiation, and who had at least 1 opioid prescription refill within the year before the end of the follow-up. We implemented the second criterion to (1) target older individuals who continued to receive prescription opioids in medical settings at which interventions could be implemented; and (2) reduce confounding by illicit opioid use, which likely occurs among ORAE cases who had no prescription opioid use before the event [19].

### Case identification

We identified ORAE cases as patients who had an inpatient or outpatient encounter with a diagnosis as defined by *International Classification of Diseases*, *Ninth or Tenth Revision*, *Clinical Modification* (*ICD-9-CM or ICD-10-CM*) codes for opioid misuse, dependence, or poisoning (**S2 Table**) [2,20]. Those codes have been used by US government agencies to define ORAEs [2,20]. Consistent with prior studies [1,19], when identifying patients with incident ORAE during follow-up, we only considered the first encounter with qualifying *ICD-10-CM* diagnosis codes. Any incident ORAE event that concurred with an injury on the same encounter was excluded to ensure temporality, that is, the injury exposure preceded an ORAE outcome. We set the date of the first eligible ORAE as the index date, and the same date was assigned as the index date to the respective matched controls.

We matched each case to 4 controls because this ratio provides optimal statistical power [21] while maintaining all eligible ORAE cases identified in Medicare data. Controls were randomly selected using incidence density sampling [22], with matching criteria including the year of cohort entry date and a disease risk score (DRS) (±0.1) for estimating future occurrence of an ORAE outcome in the absence of injury exposure during the follow-up. Incidence density sampling identifies matched controls from risk sets of patients in the cohort who were at risk but had not yet experienced an ORAE outcome at the index date of the case and who had similar matching criteria, which ensures the same length of follow-up time between cases and selected controls [22].

We used the DRS-based matching in a nested case-control setting because it allows for balancing all baseline covariates before cohort entry, increasing comparability between cases and controls, as well as yields better statistical precision than does exact matching on multiple discrete factors [23]. We calculated the DRS as the probability of predicting 2-year risk for developing an ORAE encounter during the follow-up based on prespecified risk factors (i.e., covariates measured during the 1-year pre-cohort period) via a Cox proportional hazards regression model [23,24]. The 2-year risk was predicted because the time interval is the mean follow-up of the study cohort. We utilized the “SAS macro for calculating disease risk score” created by Desia RJ [25] and computed the DRS of the ORAE outcome as the fitted values from the Cox model for each of the cohort samples (*n* = 126,752), with the exposure status set to zero (no injury).

### Measurement of injury

We examined all inpatient or outpatient medical encounters with a primary diagnosis code of injury (**S2 Table**) between prescription initiation and the index date for both cases and controls [26–28]. The validity of injury-related algorithm based on diagnosis codes for injuries in Medicare inpatient or outpatient settings is high (83.2%) [26]. Many of those types of injuries (e.g., fractures, relocation, sprains and strains, and intracranial injury) are related to falls and have been shown to be associated with opioid use [6,29–32]. When identifying patients with incident injury, we excluded *ICD-10-CM* codes that indicated a subsequent or sequela encounter. The primary exposure was the presence of incident injury at any time after opioid initiation and before the index date. The secondary exposure was recency of injury in the year pre-index period, classified into 4 categories: current (≤30 days), recent (31 to 90 days), past (91 to 180 days), and remote (181 to 365 days) injury based on the most recent diagnosis date preceding the index date. Current injury was further classified into an incident injury if there were no other diagnostic records of injury in the 31 to 365 days before the index date, otherwise, as recurrent injury. In the analysis of recency of injury, we included only case patients and controls who had at least 1 year of follow-up before the index date so that injury exposure could be assessed for a complete 1-year period for each individual. In the secondary analysis, we assessed a relationship between the cumulative number of incident injury episodes (classified as 0, 1, 2, and ≥3) in the year pre-index period and risk for ORAEs.

### Measurement of covariates

Important covariates included demographic characteristics (age, sex, and race and ethnicity), low-income subsidy status (yes versus no), US region (Northeast, Midwest, South, and West), diagnosis of alcohol or tobacco use disorder, type of chronic pain condition (musculoskeletal, neuropathic, and idiopathic), polypharmacy (having ≥5 different medications, excluding opioids), claims-based frailty index [33,34], and overall health care use (including any inpatient admission, any ED visit, and any skilled nursing facility stay). Race and ethnicity were defined based on Research Triangle Institute race code available in the Medicare claims database and were grouped into 3 groups: white, black, and other (including Hispanic, Asian, Pacific Islander, and Native American individuals), each with a sample size sufficient enough to ensure statistically reliable estimates. We also included select comorbid clinical conditions (**S2 Table**), central nervous system medications (including antipsychotics, antidepressants, anticonvulsants, benzodiazepines, non-benzodiazepines, and anxiolytics), and concurrent use of prescription opioids and benzodiazepines, all of which are strong risk factors associated with injury [35] or ORAE outcome in older adults [36]. We first measured these factors in the year before cohort entry and included them in a Cox proportional hazards regression model to estimate the DRS of developing the outcome during the follow-up, which was used for matching cases and controls. Second, clinical conditions and use of prescription opioids and central nervous system medications were treated as potential time-varying covariates, with the factors associated with the clinical condition being measured in the year pre-index period and factors associated with medication measured in the 6 months pre-index period.

### Statistical analysis

We assessed both baseline and follow-up covariate balance between cases and controls after DRS matching, with a standardized mean difference greater than 0.1 indicating covariate imbalance [37]. We used conditional logistic regression to estimate the odds ratios (ORs) and 95% confidence intervals (CIs) of ORAE risk for the main and secondary analyses, adjusting for imbalanced covariates at baseline or follow-up after matching.

We performed 3 additional analyses. First, we assessed the occurrence of injury associated with specific types of opioid encounters (i.e., opioid misuse or dependence and opioid poisoning). Second, we examined the interaction of injury and prescription opioid use within 30 days before the index date and its association with risk of ORAE. Third, we performed a sensitivity analysis using a case-crossover design to assess the association between intermittent exposure to injury events and risk of ORAEs [38]. A case-crossover study is unique in that it includes patients with ORAE only (*n* = 2,734 identified in our nested case-control study), with each patient serving as his or her own control to avoid control selection bias and measured and unmeasured time-invariant confounders. As indicated in **S1 Fig**, our exposure to injury (yes versus no) was compared between the case period (day 1 to 30) and control window (day 91 to 120) before the ORAE outcome at individual case levels. To ensure each eligible sample had complete observation of injury exposure in both risk and control windows, we included patients to those who had at least 120 days of follow-up before their ORAEs. This criterion resulted in a sample of 2,436 ORAE cases included in the case-crossover study. We used conditional logistic regression to estimate the OR of injury occurrence in the risk versus control periods, adjusting for measured covariates that differed between these 2 periods (**S6 Table**).

All analyses were performed from January 1 to December 31, 2021, using SAS, version 9.4 (SAS Institute), and all tests were 2-sided with statistical significance set as *P* < 0.05.

## Results

The study cohort consisted of 126,752 older Medicare beneficiaries with chronic noncancer pain who were new users of prescription opioids and free of injury and ORAEs in the year before cohort entry (**Fig 1**). During a mean follow-up of 1.8 years, we identified 2,734 older patients who had an incident ORAE diagnosed in inpatient or outpatient settings, yielding a rate of 12.7 per 1,000 person-years, and 10,936 matched controls for the main analysis. Among the cases and controls, 68.0% (*n* = 1,859 for cases and *n* = 7,436 for controls) were women and the mean (SD) age was 74.5 (6.9) years (Table 1).

**Table 1:** Clinical and demographic characteristics of case patients and matched controls.

	At baseline	No. (%)^a^		Follow-up	No. (%)^b^	
**Characteristic**	Cases (*n* = 2,734)	Controls (*n* = 10,936)	SDiff ^c^	Cases (*n* = 2,734)	Controls (*n* = 10,936)	SDiff^c^
**Age, y**						
Mean (SD)	74.5 (6.9)	74.5 (6.9)	<0.001	74.5 (6.9)	74.5 (6.9)	<0.001
65–74	1,449 (53.0)	5,807 (53.1)		1,449 (53.0)	5,807 (53.1)	
75–84	807 (29.5)	3,095 (28.3)		807 (29.5)	3,095 (28.3)	
≥85	478 (17.5)	1,958 (17.9)		478 (17.5)	1,958 (17.9)	
**Female**	1,856 (67.9)	7,436 (68.0)	0.004	1,856 (67.9)	7,436 (68.0)	0.004
**Race and ethnicity**			0.004			0.004
Black	284 (10.4)	1,181 (10.8)		284 (10.4)	1,181 (10.8)	
White	2,179 (79.7)	8,683 (79.4)		2,179 (79.7)	8,683 (79.4)	
Other^d^	271 (9.9)	1,072 (9.8)		271 (9.9)	1,072 (9.8)	
**Receiving low-income subsidy**	820 (30.0)	3,193 (29.2)	0.018	820 (30.0)	3,193 (29.2)	0.018
**US region**			0.020			0.020
South	1,239 (45.3)	5,063 (46.3)		1,239 (45.3)	5,063 (46.3)	
Northeast	377 (13.8)	1,378 (12.6)		377 (13.8)	1,378 (12.6)	
Midwest	571 (20.9)	2,395 (21.9)		571 (20.9)	2,395 (21.9)	
West	547 (20.0)	2,100 (19.2)		547 (20.0)	2,100 (19.2)	
**Tobacco or alcohol use disorder**	314 (11.5)	1,334 (12.2)	0.020	528 (19.3)	1,673 (15.3)	0.106
**Drug use disorder**	60 (2.2)	241 (2.2)	0.003	130 (4.8)	163 (1.5)	0.170
**Chronic pain diagnosis**						
Musculoskeletal	2,463 (90.1)	9,875 (90.3)	0.008	2,668 (97.6)	10,214 (93.4)	0.200
Neuropathic	380 (13.9)	1,553 (14.2)	0.002	1,783 (65.2)	5,654 (51.7)	0.277
Idiopathic	1,228 (44.9)	4,899 (44.8)	0.011	1,321 (48.3)	2,953 (27.0)	0.463
**Clinical condition**						
Mental health disorder	954 (34.9)	3,806 (34.8)	0.001	1,318 (48.2)	4,396 (40.2)	0.162
Diabetes	1,233 (45.1)	4,976 (45.5)	0.007	1,389 (50.8)	5,348 (48.9)	0.040
Cardiovascular disease	1,602 (58.6)	6,343 (58.0)	0.013	1,994 (71.1)	7,087 (64.8)	0.137
Hypertension	2,217 (81.1)	8,869 (81.1)	<0.001	2,392 (87.5)	9,317 (85.2)	0.068
Pulmonary condition	1,783 (65.2)	7,076 (64.7)	0.010	2,042 (74.7)	7,601 (69.5)	0.116
Kidney disease	637 (23.3)	2,570 (23.5)	0.004	1,001 (36.6)	3,445 (31.5)	0.107
Gastrointestinal tract disorder	853 (31.2)	3,292 (30.1)	0.006	1,296 (47.4)	4,035 (36.9)	0.215
Liver disease	241 (8.8)	962 (8.8)	0.023	372 (13.6)	1,126 (10.3)	0.103
Respiratory infection	927 (33.9)	3,642 (33.3)	0.013	1,110 (40.6)	3,926 (35.9)	0.096
Infection due to nonsterile opioid injection	282 (10.3)	1,115 (10.2)	0.005	448 (16.4)	1,433 (13.1)	0.094
Cognitive impairment	186 (6.8)	766 (7.0)	0.007	405 (14.8)	1,323 (12.1)	0.077
**Frailty index**						
Mean (SD)	0.17 (0.05)	0.17 (0.06)	0.039	0.21 (0.07)	0.19 (0.07)	0.224
**Polypharmacy**	2,338 (85.5)	9,427 (86.2)	0.018	2,649 (96.9)	10,499 (96.0)	0.019
**Health care use**						
Any hospital stay	470 (17.2)	1,837 (16.8)	0.010	1,055 (38.6)	3,204 (29.3)	0.079
Any ED visit	719 (26.3)	2,767 (25.3)	0.022	1,326 (48.5)	4,145 (37.9)	0.197
Any SNF stay	96 (3.5)	394 (3.6)	0.005	328 (12.0)	853 (7.8)	0.138
**Use of CNS medications**						
Benzodiazepine	525 (19.2)	2,242 (20.5)	0.031	681 (24.9)	2,264 (20.7)	0.100
Non-benzodiazepine	287 (10.5)	1,148 (10.5)	<0.001	213 (7.8)	744 (6.8)	0.040
Anticonvulsants	662 (24.2)	2,668 (24.4)	0.005	940 (34.4)	2,953 (27.0)	0.160
Antidepressants	1,074 (39.3)	4,331 (39.6)	0.008	1,148 (42.0)	4,145 (37.9)	0.085
Antipsychotics	148 (5.4)	580 (5.3)	0.003	170 (6.2)	558 (5.1)	0.047
Anxiolytics	768 (28.1)	3,193 (29.2)	0.022	842 (30.8)	2,854 (26.1)	0.100
**Prescription opioid**						
Chronic opioid use (≥90 days)	-	-	-	839 (30.7)	2,187 (20.0)	0.324
High dose (≥50 MME/daily)	-	-	-	1,042 (38.1)	2,253 (20.6)	0.451
Long-acting opioid	-	-	-	306 (11.2)	317 (2.9)	0.331
Opioid plus benzodiazepine	-	-	-	632 (23.1)	2,187 (20.0)	0.170

^a^All characteristics were measured in the year before cohort entry (i.e., opioid initiation).

^b^Diagnosis of diseases was measured in the year and use of medications was measured in the 6 months before the index date.

^c^Covariates with SDiff >0.100 represent meaningful differences between case and control groups.

^d^Included Asian, Hispanic, Native American, and Pacific Islander individuals.

CNS, central nervous system; ED, emergency department; MME, morphine milligram equivalent; SDiff, standardized difference; SNF, skilled nursing facility.

All demographic and clinical characteristics assessed were balanced before cohort entry between cases and matched controls (**Table 1**). During follow-up, some of the covariates remained balanced between cases and controls, whereas other covariates and patterns of prescription opioid use differed in that they had a standardized difference >0.100 between cases and controls, for which statistical adjustment was performed.

Overall, 54.0% (*n* = 1,475) of 2,734 cases and 43.1% (*n* = 4,718) of 10,936 controls experienced injuries between opioid initiation and the index date (**Table 2**). The adjusted odds of incident ORAE was 1.4-fold higher in patients with versus without an injury (adjusted OR [aOR] = 1.4; 95% CI 1.2 to 1.5; *P* < 0.001). Stratification analysis by specific type of opioid encounter showed higher risk of OD (aOR = 1.7; 95% CI 1.5 to 1.9; *P* < 0.001) but no difference in risk of OUD (aOR = 1.08; 95% CI 0.95 to 1.22; *P* = 0.23) among patients with versus without injury (**S3 Table**).

**Table 2:** Association between incident injury after prescription opioid initiation and subsequent risk of ORAE.

Exposure	Cases *n* = 2,734 (100%)	Controls *n* = 10,936 (100%)	Crude OR (95% CI)	*P* value	Adjusted OR^a^ (95% CI)	*P* value
*Incident injury*						
No	1,259 (46.0)	6,218 (56.9)	Reference		Reference	
Yes	1,475 (54.0)	4,718 (43.1)	1.72 (1.57–1.89)	<0.001	1.35 (1.21–1.51)	<0.001

^a^Also adjusted for imbalanced covariates at follow-up, including diagnosis of tobacco or alcohol use disorder, drug use disorder, chronic pain diagnosis, mental health disorder, cardiovascular disease, pulmonary condition, kidney disease, gastrointestinal tract disorder, liver disease, frailty index, ED visit, skilled nursing facility stay, and anticonvulsant use as well as for patterns of prescription opioid use (including use of chronic opioid use, use of high opioid dose, use of long-acting opioids, and concurrent use of opioids and benzodiazepines).

CI, confidence interval; ED, emergency department; OR, odds ratio; ORAE, opioid-related adverse event.

In the recency analysis, increased risk of ORAEs was observed for patients with current (≤30 days) injury (aOR = 2.8; 95% CI 2.3 to 3.4; *P* < 0.001) preceding the index date, whereas risk was not significant for patients with recent (31 to 90 days; aOR = 0.93; 95% CI 0.73 to 1.17; *P* = 0.48), past (91 to 180 days; aOR = 1.08; 95% CI 0.88 to 1.33; *P* = 0.51), or remote injury (181 to 365 days; aOR = 0.88; 95% CI 0.73 to 1.06; *P* = 0.18), compared with patients with no injury in the year pre-index period (**Table 3**). Of patients with current injury, those with an incident injury had a 3.8-fold risk of ORAEs (95% CI 2.9 to 4.9; *P* < 0.001) and those with recurrent injury had a 2.3-fold risk of ORAEs (95% CI 1.8 to 2.9; *P* < 0.001), compared with patients without injury. Stratification analysis by specific type of opioid encounter showed similar results, with higher risk of ORAEs detected among patients with current injury only (for OD: aOR = 4.1; 95% CI 3.2 to 5.2; *P* < 0.001; for OUD: aOR = 1.3; 95% CI 1.02 to 1.71; *P* = 0.04) (**S4 and S5 Tables)**. The finding of increased risk of ORAEs with current injury but not with past injury persisted in the case-crossover design (aOR = 2.3; 95% CI 1.8 to 3.0; *P* < 0.001) (**S7 Table)**. There were significant associations between cumulative injury episodes in the year pre-index period and risk of ORAEs in unadjusted models (OR = 1.5; 95% CI 1.3 to 1.7; *P* < 0.001 for 1 injury episode; OR = 1.6; 95% CI 1.3 to 2.0; *P* < 0.001 for 2 injury episodes; OR = 1.8; 95% CI 1.6 to 2.2; *P* < 0.001 for 3 or more injury episodes versus none). In adjusted models, the associations were attenuated but remained significant (aOR = 1.2; 95% CI 1.1 to 1.4; *P* = 0.01 for 1 injury episode; aOR = 1.3; 95% CI 1.1 to 1.6; *P* < 0.001 for 2 injury episodes; aOR = 1.3; 95% CI 1.1 to 1.5; *P* = 0.01 for 3 or more injury episodes versus none).

**Table 3:** Risk of ORAEs by recency and cumulative number of injurious episodes in the year before the index date^a^.

Injury	Cases, No (%) *n* = 1,725	Controls, No. (%) *n* = 6,900	Crude OR (95% CI)	*P* value	Adjusted OR^b^ (95% CI)	*P* value
*Recency of Injury*						
None	933 (54.1)	4,505 (65.3)	Reference		Reference	
Current (≤30 days)	298 (17.3)	407 (5.9)	3.52 (2.98–4.16)	<0.001	2.81 (2.32–3.39)	<0.001
New event	127 (7.4)	150 (2.2)	4.06 (3.17–5.20)	<0.001	3.76 (2.86–4.94)	<0.001
Recurrent event	171 (9.9)	257 (3.7)	3.20 (2.60–3.94)	<0.001	2.30 (1.82–2.90)	<0.001
Recent (31–90 days)	128 (7.4)	495 (7.2)	1.26 (1.02–1.55)	0.03	0.93 (0.73–1.17)	0.48
Past (91–180 days)	163 (9.5)	565 (8.2)	1.41 (1.17–1.71)	<0.001	1.08 (0.88–1.33)	0.51
Remote (181–365 days)	203 (11.8)	928 (13.5)	1.05 (0.88–1.24)	0.60	0.88 (0.73–1.06)	0.18
*Cumulative No*. *of injury episodes*						
None	933 (54.1)	4,505 (65.3)	Reference		Reference	
1	356 (20.6)	1,187 (17.2)	1.45 (1.26–1.66)	<0.001	1.22 (1.05–1.42)	0.01
2	175 (10.1)	522 (7.6)	1.62 (1.34–1.95)	<0.001	1.31 (1.07–1.61)	<0.001
≥3	261 (15.1)	686 (9.9)	1.84 (1.57–2.16)	<0.001	1.28 (1.06–1.54)	0.01

^a^Study sample included older patients with ≥1 year of follow-up.

^b^Also adjusted for imbalanced covariates at follow-up, including diagnosis of tobacco or alcohol use disorder, drug use disorder, chronic pain diagnosis, mental health disorder, cardiovascular disease, pulmonary condition, kidney disease, gastrointestinal tract disorder, liver disease, frailty index, ED visit, skilled nursing facility stay, and anticonvulsant use as well as for patterns of prescription opioid use (including use of chronic opioid use, use of high opioid dose, use of long-acting opioids, and concurrent use of opioids and benzodiazepines).

CI, confidence interval; ED, emergency department; OR, odds ratio; ORAE, opioid-related adverse event.

The interaction of injury and prescription opioid use within the 30 days before the index date was significantly associated with the risk of ORAEs (*P* < 0.001 for interaction) (**Table 4**). Increased risk of ORAEs was observed among older patients with both injury and prescription opioid use (aOR = 5.0; 95% CI 4.1 to 6.1; *P* < 0.001) and among older patients with injury but no prescription opioid use (aOR = 4.6; 95% CI 3.7 to 5.7; *P* < 0.001), compared with older patients with neither injury nor prescription opioid use in the month pre-index period.

**Table 4:** Interaction of injury and prescription opioid use within the 30 days before the index date and risk of ORAEs.

	Cases, No (%)	Controls, No, (%)	Crude OR (95% CI)	*P* value	Adjusted OR^b^ (95% CI)	*P* value
*Interaction of injury with opioid use* ^a^	*n* = 2,734	*n* = 10,936				
No injury and no opioid use	861 (31.5)	6,934 (63.4)	Reference		Reference	
No injury and opioid use	1,395 (51.0)	3,425 (31.3)	3.43 (3.11–3.79)	<0.001	2.32 (2.06–2.62)	<0.001
Injury and no opioid use	179 (6.6)	272 (2.5)	5.34 (4.35–6.56)	<0.001	4.55 (3.65–5.67)	<0.001
Injury and opioid use	299 (10.9)	305 (2.8)	8.28 (6.92–9.92)	<0.001	5.00 (4.06–6.14)	<0.001
***P* for interaction**						0.001

^a^Measured in the 30 days before the index date.

^b^Also adjusted for imbalanced covariates at follow-up, including diagnosis of tobacco or alcohol use disorder, drug use disorder, chronic pain diagnosis, mental health disorder, cardiovascular disease, pulmonary condition, kidney disease, gastrointestinal tract disorder, liver disease, frailty index, ED visit, skilled nursing facility stay, and anticonvulsant use as well as for patterns of prescription opioid use (including use of chronic opioid use, use of high opioid dose, use of long-acting opioids, and concurrent use of opioids and benzodiazepines).

CI, confidence interval; ED, emergency department; OR, odds ratio; ORAE, opioid-related adverse event.

## Discussion

In this sample of older Medicare beneficiaries with chronic noncancer pain, we found that injury occurring after prescription opioid initiation was independently associated with a 1.4-fold increased risk for ORAEs after adjusting for time-varying confounders. Timing of injury also mattered in estimating future ORAEs. Injuries occurring in the month preceding the index date for both cases and controls were associated with a 2.8-fold increased risk for ORAEs, whereas risk was not significantly associated with recent, past, and remote injury preceding the index date. Consistent results were found in an alternative case-crossover design and across subgroup analyses. Our findings indicated that the presence of injury after initial use of prescription opioid therapy may be a warning signal suggestive of future incident ORAEs among older opioid users.

Our findings add to the existing research by supporting the previously raised hypothesis [8,9,39] that injury may be an indicator that emerges between prescription opioid initiation and ORAEs among older adults. Although prior studies have shown an increased risk for injuries (including falls, fractures, car crashes, head, and traumatic injuries) associated with opioid use [6,29–31], none have explored a prognostic role of injury associated with ORAEs among older opioid users. Injury may reflect potential opioid misuse and use disorder developed over time prior to the injury [7,39], which is especially challenging to identify among older adults because many opioid misuse symptoms (e.g., sudden memory loss and confusion) mimic common geriatric symptoms [30]. Yet, aberrant opioid use behaviors, early precursors of ORAEs, have been shown to impair older individuals’ cognitive and psychomotor functions, leading to increased risk for unintentional injury [8]. Opioid-related injuries without timely interventions may increase chronic opioid use, further heightening the chance of another injury and leading to the downstream consequence of ORAEs [40].

Our finding also adds to the existing research by showing a high OR (5.0) for ORAE in older individuals with both injury and prescription opioid use versus patients with neither in the month pre-index period deserves attention. This observation highlights the potential importance of both factors when simultaneously occurring in the month pre-index period may have a synergistic association with an increased risk of ORAE among older patients. We also observed a significantly increased risk of ORAEs (4.6-fold) in the subgroup of older patients who had an injury but had no prescription opioid use (versus those with neither) in the month pre-index period. Increased risk in this subgroup may be attributable to improper use of opioids prescribed prior to the last month before the index date or to an OUD that has developed over time. Alternatively, this subgroup with an injury but no prescription opioids might have proper use of their previously prescribed opioids and the increased subsequent ORAE risk could be reflective of potential illicit opioid use before or during the last month.

A notable strength of our study is the use of a nationally representative sample of older adults in a Medicare sample of recent years. Those national data provided a sufficient number of older adults with incident ORAEs, allowing for adequate power to detect the association between injury and risk for ORAEs. The study also had limitations. First, residual confounding caused by unknown or unmeasured confounders is possible. For example, illicit opioid use, which cannot be captured in Medicare data, is a confounder associated with injury and contributes to ORAEs among older adults. As well, Medicare data have no information on alcohol and smoking behavior, and we mitigated this limitation to some extent by using the diagnosis of alcohol and tobacco use disorder as a proxy. Second, we are unable to determine whether injury was caused by opioid misuse or other reasons (e.g., uncontrolled pain). Third, not all fatal injuries would be present in inpatient or outpatient care, and we included only injuries that received medical attention. Thus, we could not rule out the possibility of exposure misclassification (i.e., patients were misclassified as having no injury because their events did not present in medical settings). Fourth, while OD defined in inpatient claims data has been validated against chart review, with a high positive predictive value (81%) [41], the validity of ORAEs, which includes both OD and OUD defined in inpatient and outpatient claims, is unclear and warrants further research. A suboptimal accuracy of the administrative data in detecting OUD cases is observed largely due to the underdiagnosis of the condition [42]. The underdiagnosis of OUD, if differential by injury status, may potentially bias our estimated association. Fifth, our findings can be generalized only to Medicare fee-for-service beneficiaries. Finally, our findings derived from 2011 to 2018 data may not be reflective of clinical practices after 2018, during which patterns of opioid prescribing and its relevance to ORAEs may have been changed.

Our findings have important clinical implications for older adults who are prescribed opioids for pain. Identification of injuries that emerge between opioid initiation and ORAEs may assist health care professionals in the early detection of older opioid users at high risk for OUD or OD. Such prevention can be implemented within the existing clinical protocol for opioid prescribing by retrospectively reviewing patient electronic health records for inpatient or outpatient encounters of injuries that occurred before their clinical visits. These injury events may serve as a warning to alert clinicians that older patients may have already engaged in opioid misuse, promoting further risk assessment and caution in prescribing opioids to prevent older opioid users from progressing toward more severe opioid-related harms, such as opioid overdose and death.

## Conclusion

In this sample of older patients who are Medicare beneficiaries, incident injury after prescription opioid therapy was associated with subsequent increased risk of ORAEs, and the risk was only significant among patients with injury in the month before the index date. Our findings suggest that regular monitoring of injury events after initiating prescription opioids may help identify older opioid users at risk for ORAEs.

## Supporting information

S1 STROBE Checklist Strengthening the reporting of observational studies in epidemiology (STROBE) checklist. (DOC)

S1 Text Prespecified analysis plan. (DOCX)

S1 Table Study prescription opioids approved by the US Food and Drug Administration for use in the US market from 2011 to 2018. (DOCX)

S2 Table *ICD-9-CM*, *ICD-10-CM*, or E codes and procedures for disease conditions and service care considered in the study. (DOCX)

S3 Table Association between incident injury after prescription opioid initiation and subsequent risk of opioid overdose and risk of opioid use disorder. (DOCX)

S4 Table Risk of opioid overdose by recency of injury in the year before the index date among older patients with ≥1 year of follow-up. (DOCX)

S5 Table Risk of opioid use disorder by recency of injury in the year before the index date among older patients with ≥1 year of follow-up. (DOCX)

S6 Table Characteristics of patients with ORAEs during the risk and control periods in a case-crossover study design. (DOCX)

S7 Table Findings of case-crossover analyses of the association between injury and risk of opioid-related adverse events. (DOCX)

S1 Fig Specification of risk and control windows in a case-crossover design. (TIFF)

## Abbreviations

aOR: adjusted odds ratio
CI: confidence interval
CMS: Centers for Medicare and Medicare Services
DRS: disease risk score
ED: emergency department
OD: opioid overdose
OR: odds ratio
ORAE: opioid-related adverse event
OUD: opioid use disorder

## References

[1] Wei YJ, Chen C, Schmidt SO, LoCiganic WH, Winterstein AG. Trends in prior receipt of prescription opioid or adjuvant analgesics among patients with incident opioid use disorder or opioid-related overdose from 2006 to 2016. Drug Alcohol Depend. 2019;204:107600. doi: 10.1016/j.drugalcdep.2019.107600 31586806 PMC6927577

[2] Agency for Healthcare Research and Quality. Opioid-Related Inpatient Stays and Emergency Department Visits Among Patients Aged 65 Years and Older, 2010 and 2015. Available from: https://www.hcup-us.ahrq.gov/reports/statbriefs/sb244-Opioid-Inpatient-Stays-ED-Visits-Older-Adults.jsp. 30475561

[3] The Substance Abuse and Mental Health Services Administration. Opioid misuse increases among older adults. 2017. Available from: https://www.samhsa.gov/data/sites/default/files/report_3186/Spotlight-3186.html.

[4] McBain R, Rose AJ, LaRochelle MR. The U.S. opioid epidemic: one disease, diverging tales. Prev Med. 2018;112:176–178. doi: 10.1016/j.ypmed.2018.04.023 29684417

[5] Schieber LZ, Guy GP Jr, Seth P, Losby JL. Variation in adult outpatient opioid prescription dispensing by age and sex—United States, 2008–2018. MMWR Morb Mortal Wkly Rep. 2020;69(11):298–302. doi: 10.15585/mmwr.mm6911a5 32191686 PMC7739983

[6] Rubin R. Opioid-Related Problems Increasing Among Older Adults. JAMA. 2018;320(20):2067. doi: 10.1001/jama.2018.17630 30480710

[7] Basca B. The Elderly and Prescription Drug Misuse and Abuse. 2017. Available from: http://www.cars-rp.org/wp-content/uploads/2014/06/Prevention-Tactics-Vol09-No02-2008.pdf.

[8] Carter MW, Yang BK, Davenport M, Kabel A. Increasing rates of opioid misuse among older adults visiting emergency departments. Innov Aging. 2019;3(1):igz002. doi: 10.1093/geroni/igz002 30863796 PMC6404687

[9] Alghnam S, Castillo R. Traumatic injuries and persistent opioid use in the USA: findings from a nationally representative survey. Inj Prev. 2017;23(2):87–92. doi: 10.1136/injuryprev-2016-042059 27597400

[10] Dunn KM, Saunders KW, Rutter CM, Banta-Green CJ, Merrill JO, Sullivan MD, et al. Opioid prescriptions for chronic pain and overdose: a cohort study. Ann Intern Med. 2010;152(2):85–92. doi: 10.7326/0003-4819-152-2-201001190-00006 20083827 PMC3000551

[11] Chou R, Turner JA, Devine EB, Hansen RN, Sullivan SD, Blazina I, et al. The effectiveness and risks of long-term opioid therapy for chronic pain: a systematic review for a National Institutes of Health Pathways to Prevention Workshop. Ann Intern Med. 2015;162(4):276–286. doi: 10.7326/M14-2559 25581257

[12] Services. TCfMaM. Medicare Program—General Information. Available from: https://www.cms.gov/Medicare/Medicare-General-Information/MedicareGenInfo.

[13] (CMS) TCfMaMS. CMS Program Statistics: Medicare Enrollment. Available from: https://www.cms.gov/research-statistics-data-systems/cms-program-statistics/medicare-enrollment.

[14] Center RDA. Enhanced Medicare 5% Sample Indicator. Available from: https://resdac.org/cms-data/variables/enhanced-medicare-5-sample-indicator.

[15] Baron JA, Lu-Yao G, Barrett J, McLerran D, Fisher ES. Internal validation of Medicare claims data. Epidemiology. 1994;5(5):541–544. 7986870

[16] Weintraub WS, Bellows BK. Evaluating Clinical Outcomes From Administrative Databases. JACC Cardiovasc Interv. 2020;13(15):1786–1788. doi: 10.1016/j.jcin.2020.04.023 32682675 PMC7848782

[17] Leonard CE, Brensinger CM, Nam YH, Bilker WB, Barosso GM, Mangaali MJ, et al. The quality of Medicaid and Medicare data obtained from CMS and its contractors: implications for pharmacoepidemiology. BMC Health Serv Res. 2017;17(1):304. doi: 10.1186/s12913-017-2247-7 28446159 PMC5406992

[18] Schneeweiss S, Suissa S. Discussion of Schuemie et al: “A plea to stop using the case-control design in retrospective database studies”. Stat Med. 2019;38(22):4209–4212. 31489683 10.1002/sim.8320

[19] Wei YJ, Chen C, Fillingim R, Schmidt SO, Winterstein AG. Trends in prescription opioid use and dose trajectories before opioid use disorder or overdose in US adults from 2006 to 2016: A cross-sectional study. PLoS Med. 2019;16(11):e1002941. doi: 10.1371/journal.pmed.1002941 31689302 PMC6830744

[20] Rajbhandari-Thapa J, Zhang D, Padilla HM, Chung SR. Opioid-Related Hospitalization and Its Association With Chronic Diseases: Findings From the National Inpatient Sample, 2011–2015. Prev Chronic Dis. 2019;16:E157. doi: 10.5888/pcd16.190169 31775008 PMC6896831

[21] Rothman KJ. Modern epidemiology. Boston Little BaC, editor. 1986.

[22] Richardson DB. An incidence density sampling program for nested case-control analyses. Occup Environ Med. 2004;61(12):e59. doi: 10.1136/oem.2004.014472 15550597 PMC1740694

[23] Desai RJ, Glynn RJ, Wang S, Gagne JJ. Performance of Disease Risk Score Matching in Nested Case-Control Studies: A Simulation Study. Am J Epidemiol. 2016;183(10):949–957. doi: 10.1093/aje/kwv269 27189330

[24] Arbogast PG, Ray WA. Use of disease risk scores in pharmacoepidemiologic studies. Stat Methods Med Res. 2009;18(1):67–80. doi: 10.1177/0962280208092347 18562398

[25] Desai RJ, Wyss R, Jin Y, Bohn J, Toh S, Cosgrove A, et al. Extension of Disease Risk Score-Based Confounding Adjustments for Multiple Outcomes of Interest: An Empirical Evaluation. Am J Epidemiol. 2018;187(11):2439–2448. doi: 10.1093/aje/kwy130 29947726

[26] Min L, Tinetti M, Langa KM, Ha J, Alexander N, Hoffman GJ. Measurement of Fall Injury With Health Care System Data and Assessment of Inclusiveness and Validity of Measurement Models. JAMA Netw Open. 2019;2(8):e199679. doi: 10.1001/jamanetworkopen.2019.9679 31433480 PMC6707014

[27] Hedegaard H, Johnson RL, Garnett MF, Thomas KE. The 2020 International Classification of Diseases, 10th Revision, Clinical Modification Injury Diagnosis Framework for Categorizing Injuries by Body Region and Nature of Injury. Natl Health Stat Report. 2020;150:1–27. 33395385

[28] Recommended framework for presenting injury mortality data. MMWR Recomm Rep. 1997;46(RR-14):1–30. 9301976

[29] Buckeridge D, Huang A, Hanley J, Kelome A, Reidel K, Verma A, et al. Risk of injury associated with opioid use in older adults. J Am Geriatr Soc. 2010;58(9):1664–70. doi: 10.1111/j.1532-5415.2010.03015.x 20863326

[30] Miller M, Sturmer T, Azrael D, Levin R, Solomon DH. Opioid analgesics and the risk of fractures in older adults with arthritis. J Am Geriatr Soc. 2011;59(3):430–438. doi: 10.1111/j.1532-5415.2011.03318.x 21391934 PMC3371661

[31] Li G, Chihuri S. Prescription opioids, alcohol and fatal motor vehicle crashes: a population-based case-control study. Inj Epidemiol. 2019;6:11. doi: 10.1186/s40621-019-0187-x 31245260 PMC6582661

[32] Padmanathan P, Forbes H, Redaniel MT, Gunnell D, Lewer D, Moran P, et al. Self-harm and suicide during and after opioid agonist treatment among primary care patients in England: a cohort study. Lancet Psychiatry. 2022;9(2):151–159. doi: 10.1016/S2215-0366(21)00392-8 34921800

[33] Kim DH, Schneeweiss S, Glynn RJ, Lipsitz LA, Rockwood K, Avorn J. Measuring Frailty in Medicare Data: Development and Validation of a Claims-Based Frailty Index. J Gerontol A Biol Sci Med Sci. 2018;73(7):980–987. doi: 10.1093/gerona/glx229 29244057 PMC6001883

[34] Gautam N, Bessette L, Pawar A, Levin R, Kim DH. Updating International Classification of Diseases 9th Revision to 10th Revision of a Claims-Based Frailty Index. J Gerontol A Biol Sci Med Sci. 2021;76(7):1316–1317. doi: 10.1093/gerona/glaa150 32529241 PMC8202141

[35] Johnell K, Jonasdottir Bergman G, Fastbom J, Danielsson B, Borg N, Salmi P. Psychotropic drugs and the risk of fall injuries, hospitalisations and mortality among older adults. Int J Geriatr Psychiatry. 2017;32(4):414–420. doi: 10.1002/gps.4483 27113813 PMC5347947

[36] Reid MC, Eccleston C, Pillemer K. Management of chronic pain in older adults. BMJ. 2015;350:h532. doi: 10.1136/bmj.h532 25680884 PMC4707527

[37] Austin PC. Balance diagnostics for comparing the distribution of baseline covariates between treatment groups in propensity-score matched samples. Stat Med. 2009;28(25):3083–3107. doi: 10.1002/sim.3697 19757444 PMC3472075

[38] Maclure M, Mittleman MA. Should we use a case-crossover design? Annu Rev Public Health. 2000;21:193–221. doi: 10.1146/annurev.publhealth.21.1.193 10884952

[39] Bebinger M. What Doesn’t Kill You Can Maim: Unexpected Injuries From Opioids. National Public Radio. 2017.

[40] Daoust R, Paquet J, Moore L, Emond M, Gosselin S, Lavigne G, et al. Recent opioid use and fall-related injury among older patients with trauma. CMAJ. 2018;190(16):E500–E506. doi: 10.1503/cmaj.171286 29685910 PMC5915247

[41] Green CA, Perrin NA, Janoff SL, Campbell CI, Chilcoat HD, Coplan PM. Assessing the accuracy of opioid overdose and poisoning codes in diagnostic information from electronic health records, claims data, and death records. Pharmacoepidemiol Drug Saf. 2017;26(5):509–517. doi: 10.1002/pds.4157 28074520

[42] Palumbo SA, Adamson KM, Krishnamurthy S, Manoharan S, Beiler D, Seiwell A, et al. Assessment of Probable Opioid Use Disorder Using Electronic Health Record Documentation. JAMA Netw Open. 2020;3(9):e2015909. doi: 10.1001/jamanetworkopen.2020.15909 32886123 PMC7489858

