# Supplementary material for: Association of injury after prescription opioid initiation with risk for opioid-related adverse events among older Medicare beneficiaries in the United States: A nested case-control study
Source: PLoS Med. 2022 Sep 22;19(9):e1004101. doi: 10.1371/journal.pmed.1004101 (PMC9498946; doi:10.1371/journal.pmed.1004101)
Supplement: S1 Text — (DOCX) [file pmed.1004101.s002.docx]

**Study Aims:** The primary aim was to examine the association between incident injury after prescription opioid initiation and the risk of ORAEs. The secondary aim was to assess whether the association differs by recency of injury among older patients

**Research design:** Nested case-control study to include all identified ORAE cases, which are relatively rare among older adults compared with younger populations^1^ and to study the association between recency of injury exposure and risk for ORAEs. We also use a case-crossover study design as a sensitivity analysis to assess recency of injury and risk for ORAEs.

**Data source:** 5% random national sample of Medicare fee-for-service (FFS) beneficiaries from 2011- to 2018 will constitute the study population.

**Sample selection:**

*Step 1: create an opioid-naïve cohort by applying the following criteria:*

1. Initiated prescription opioids during 1/1/2012-12/31/2018
   1. Opioid initiation is defined as no opioid prescription dispensed within 12 months preceding the date of the first opioid prescription fill (i.e., “opioid initiation date”).
2. Aged ≥65 years on the opioid initiation date
3. Had at least one of these three chronic pain conditions: a) musculoskeletal pain; b) neuropathic pain; or c) idiopathic pain diagnosed within 12 months before the opioid initiation date
4. Had survived throughout 12 months before the opioid initiation date
5. Had continuously enrolled in Parts A/B/PDP and had no HMO/EMP coverage throughout the 12 months before the opioid initiation date
6. Had no cancer, hospice care, and palliative care during the 12 months before the opioid initiation date
7. Had no diagnosis of injury (exposure of interest) during the 12 months before or on the opioid initiation date
8. Had no opioid-related adverse events (ORAE, i.e., outcome of interest) during the 12 months before the opioid initiation date

*Follow-up*: From opioid initiation date until an ORAE event, cancer diagnosis, receiving hospice care, death, Medicare disenrollment, or study end (12/31/2018)

*Step 2: select cases and controls from the cohort created in step 1*

1. Select ***incident* cases** who
   1. had an ORAE during the follow-up, and
   2. Exclude ICD-10-CM codes of opioid-related outcome events indicating opioid dependence or abuse “in remission”
   3. Exclude cases that had less than 30 days of follow-up because of the insufficient period for measuring incident injury (key exposure of interest)
2. For each case, randomly select ***4 controls*** using incidence-density sampling and match on the year of cohort entry date and a disease risk score (see detailed method in **S2 Text**)
   1. Assign the date of matched controls on the event date for the case

Present sample selection in a flow chart (i.e., Figure 1)

*Step 3:* For analysis of recency of injury, restricted the case patients and controls identified from Step 2 to those who had at least 1 year of follow-up before the index date so that injury exposure could be assessed for a complete 1-year period for each individual

**Injury** (*key exposure*): We examined all inpatient or outpatient medical encounters with a primary diagnosis code of injury (**S2 Table**) between prescription initiation and the index date for both cases and controls. In the identification of an incident injury diagnosis, we required patients to have at least one inpatient, skilled nursing facility, home health agency, hospice outpatient, or carrier claim with disease code in primary diagnostic position. To ensure the identification of incident injury, when using ICD-10-CM codes, we included only xx. x[xxxA](https://www.icd10data.com/ICD10CM/Codes/S00-T88/S00-S09/S01-/S01.00XA), xx.xxxxB, or xx. xxxxC (initial encounter).

The secondary exposure was recency of injury in the year pre-index period, classified into 4 categories: current (≤30 days), recent (31-90 days), past (91-180 days), and remote (181-365 days) injury based on the most recent diagnosis date preceding the index date. Current injury was further classified into an incident injury if there were no other diagnostic records of injury in the 31 to 365 days before the index date; otherwise, as recurrent injury.

**Covariates measured in the study are detailed as followings:**

1. *Demographics*: age (65-74, 75-84, 85+), sex, race/ethnicity (Whites, Blacks, and Others), low-income subsidy status (Yes vs no), place of residence (based on five-digit ZIP codes and classified as South, Northeast, Midwest, and West), all of which are derived from Medicare Beneficiary Summary File.
2. *Health status*: Tobacco or alcohol use disorder (Yes vs no, assessed based on the diagnostic algorithm for both conditions developed by the Chronic Condition Warehouse, <https://www2.ccwdata.org/web/guest/condition-categories>) and drug use disorder.
3. *Chronic pain diagnosis*: classified as musculoskeletal pain (Yes vs no), neuropathic pain (Yes vs no), or idiopathic pain (Yes vs no). See ICD-9 or ICD-10 codes in the supplemental file. Note that an individual can have more than one type of chronic pain conditions.
4. *Clinical conditions* that may affect opioid treatment, include mental health disorders, diabetes, cardiovascular diseases, hypertension, pulmonary condition, kidney disease, gastrointestinal tract disorder, liver disease, respiratory infections, infections due to nonsterile opioid injection, and cognitive impairment
5. *Health care utilization*: any hospital stay (yes vs no), any emergency department visit (yes vs no), any skilled nursing facility stay (yes vs no)
6. *Medication*-*related factors*: polypharmacy (defined as the use of > 4 distinct generic drugs simultaneously), use of central nervous system medications (including benzodiazepine, non-benzodiazepine, anticonvulsants, antidepressants, antipsychotics, anxiolytics), prescription opioid use (dose in daily morphine milligram equivalents, long-active, concurrent use of opioids and benzodiazepine).

We measured these covariates in two different periods. First, we measured them during the 12 months before cohort entry (opioid initiation date) and included them in a Cox proportional hazards regression model to estimate the DRS of developing the outcome during the follow-up, which was used for matching cases and controls. Second, we measured them during follow-up and treated them as potential time-varying covariates. We measured factors associated with clinical condition and health care utilization in the year pre-index period and factors associated with medication measured in the 6 months pre-index period.

**Data analyses:** Our overall analytic approach include descriptive analyses, group-based trajectory modeling, and multivariable conditional logistic models. All tests are at two-sided with a statistical significance of P < .05. Our analytical plan is detailed as follows:

1. Descriptive analysis:

Table 1: Describe baseline and follow-up characteristics of Clinical and Demographic Characteristics of Case Patients and Matched Controls

1. Multivariable conditional logistic models:

Table 2: Association Between Incident Injury After Prescription Opioid Initiation and Subsequent Risk of Opioid-Related Adverse Event

Table 3: Risk of Opioid-Related Adverse Events by Recency and Cumulative Number of Injurious Episodes in the Year Before the Index Date

Table 4. Interaction of Injury and Prescription Opioid Use Associated With the Risk of Opioid-Related Adverse Events

Sensitivity analysis:

Supplement text: A Case-Crossover Study Design for Assessing the Association Between Injury and Risk of Opioid-Related Adverse Events

Supplemental tables:

S3 Table. Association Between Incident Injury After Prescription Opioid Initiation and Subsequent Risk of OUD and Risk of OD

S4 Table. Findings of Risk of OD by Recency of Injury in the Year Before the Index Date

S5 Table. Findings of Risk of OUD by Recency of Injury in the Year Before the Index Date

S6 Table. Findings of Case-Crossover Analyses of the Association Between Injury and Risk of Opioid-Related Adverse Events
