# Supplementary material for: Association of injury after prescription opioid initiation with risk for opioid-related adverse events among older Medicare beneficiaries in the United States: A nested case-control study
Source: PLoS Med. 2022 Sep 22;19(9):e1004101. doi: 10.1371/journal.pmed.1004101 (PMC9498946; doi:10.1371/journal.pmed.1004101)
Supplement: S2 Table — (DOCX) [file pmed.1004101.s004.docx]

**S2 Table.** *ICD-9-CM, ICD-10-CM*, or E Codes and Procedures for Disease Conditions and Service Care Considered in the Study

| **Disease, Condition, or Service Care** | ***ICD-9-CM, ICD-10-CM*, or E code or procedure** | **Algorithm** |
| --- | --- | --- |
| Opioid-related adverse event | Opioid misuse or dependence (ICD-9)  305.50 (Opioid abuse – unspecified)  305.51 (Opioid abuse – continuous)  305.52 (Opioid abuse – episodic)  304.00 (Opioid type dependence – unspecified)  304.01 (Opioid type dependence – continuous)  304.02 (Opioid type dependence – episodic)  304.70 (Combinations of opioid type drug with any other – unspecified)  304.71 (Combinations of opioid type drug with any other – continuous)  304.72 (Combinations of opioid type drug with any other – episodic) | At least 1 inpatient, SNF, HHA, or carrier claim with disease code in any diagnostic position |
|  | Opioid poisoning (ICD-9):  965.00 (Poisoning – opium (alkaloids), unspecified)  965.01 (Poisoning – heroin)  965.02 (Poisoning – methadone)  965.09 (Poisoning – opiates and related narcotics, other)  E850.0 (Accidental poisoning by heroin)  E850.1 (Accidental poisoning by methadone)  E850.2 (Accidental poisoning by other opiates and related narcotics) |  |
|  | Corresponded ICD-10 codes for opioid misuse, opioid dependence, and opioid poisoning are listed at https://www.hcup-us.ahrq.gov/reports/statbriefs/sb258-Opioid-Hospitalizations-Rural-Metro-Hospitals-2016.jsp |  |
| Injury | *ICD-9-CM* codes  800-829 (Fracture)  830-839 (Dislocation)  840-848 (Sprains and Strains of joints and adjacent muscles)   - 1. (Intracranial injury, excluding those with skull fracture)   860-869 (Internal injury of chest, abdomen, and pelvis)  870-894 (Open wound)  900-904 (Injury to blood vessels)  910-919 (Superficial injury)  920-929 (Contusion, crushing injury)  940-949 (Burns)  950-957 (Injury to nerves and spinal cord)  958-959 (Certain traumatic complications and unspecified injuries) | For exclusion criterion: At least 1 inpatient, SNF, HHA, HOP, or carrier claim with disease code in any diagnostic position.  For identification of an incident injury: At least 1 inpatient, SNF, HHA, HOP, or carrier claim with disease code in primary diagnostic position. |
|  | *ICD-10-CM code*  S00-S09 (Injuries to the head)  S10-S19 (Injuries to the neck)  S20-S29 (Injuries to the thorax)  S30-S39 (Injuries to the abdomen, lower back, lumbar spine, pelvis, and external genitals)  S40-S49 (Injuries to the shoulder and upper arm)  S50-S59 (Injuries to the elbow and forearm))  S60-S69 (Injuries to the wrist, hand, and fingers)  S70-S79 (Injuries to the hip and thigh)  S80-S89 (Injuries to the knee and lower leg)  S90-S99 (Injuries to the ankle and foot)  T07 (Injuries involving multiple body regions)  T14 (Injury of unspecified body region)  T20-T25(Burns and corrosions of the external body surface, specified by site)  T26-T28 (Burns and corrosions confined to eye and internal organs)  T30-T32 (Burns and corrosions of multiple and unspecified body regions)  ¶ For an incident injury, only xx. x[xxxA](https://www.icd10data.com/ICD10CM/Codes/S00-T88/S00-S09/S01-/S01.00XA), xx.xxxxB, or xx. xxxxC (initial encounter) were used |  |
| Chronic pain |  |  |
| Musculoskeletal | 274.x, 710.x-729.x (exclude 723.4, 724.3, 724.4, 729.1, 729.2), A18.01-A18.02, A52.16, D48.1, E08.61x, E09.61x, E10.61x, E11.61x, E13.61x, M00-M02, M04.02-M04.09, M05-M19, M1A, M20.10, M21.61-M21.62, M22-M25, M32-M36, M43.2-M43.8X9, M45-M48, M49.80, M50, M51, M53, M54, M60.0-M60.2, M61-M63, M65-M67, M70-M72, M75-M77, M79, M96.1, M99.2-M99.7, N20.0, Q68.6, R25.2, R26.2, R29.8x | At least 1 inpatient, SNF, HHA, HOP, or carrier claim with disease code in any diagnostic position |
| Neuropathic | 053.1x, 249.6, 250.6, 307.89, 336.x, 337.x, 338.0, 340, 350.x, 351.x, 352.1, 353.x-355.x, 357.1, 357.2-357.4, 357.8, 357.9, 723.4, 724.3, 724.4, 729.1, 729.2,  A52.15, B02 (exclude B02.1), EXX.4, EXX.610, EXX.65 (where X in “08”-“13”), E10.4, F45.42, G13.0, G13.1, G32.0, G35, G50- G52.1, G54-G59, G61.8, G61.9, G62.8, G62.9, G63-G65, G89.0, G90.0, G90.5, G95, G99.0-G99.2, M05.5, M54.13-M54.18, M54.3, M54.4, M60.8, M60.9, M79.1, M79.2, M79.7 |  |
| Idiopathic | 338.2, 338.4, 780.96, G89, R52 |  |
| Cancer diagnosis | CCS11-CCS43 | HCUP CCS for *ICD-9-CM or ICD-10-CM* |
| Hospice care | Admission date of hospice claims | At least 1 hospice claim in any diagnostic position |
| Palliative Care | DX: V 66.7  Provide specialty code: 17 | At least 1 inpatient, SNF, HHA, HOP, carrier, or DME claim with disease code in any diagnostic position; or at least 1 inpatient, SNF, HHA, HOP with provider specialty code |
| Tobacco use | 305.1, 649.0x, 989.84,F17.x, O99.33x, T65.21xA, Z72.0 | At least 1 inpatient, SNF, HHA, HOP, carrier, or DME claim with disease code in any diagnostic position |
| Alcohol use disorder | 291.x, 303.x, 305.0x, 357.5, 425.5, 535.3x, 571.0-571.3, 760.71, 980.0, V65.42, V79.1, E860.0, F10.x (excluding F10.11, F10.13, F10.21, F10.93), G62.1, I42.6, K29.2x, K70.x, P04.3, Q86.0, T51.0XxA, Z71.4x |  |
| Drug use disorder | DX 292.x, 304.x (excluding 304.00, 304.01, 304.02, 304.70, 304.71, 304.72), 305.x (excluding 305.0x, 305.1, 305.5x), 648.3, 648.30, 648.31, 648.32, 648.33, 648.34, 655.5, 655.50, 655.51, 655.53, 760.72, 760.73, 760.75, 779.5, V65.42, E854.1. | At least 1 inpatient, SNF, HHA, HOP, carrier, or DME claim with disease code in any diagnostic position |
|  | Corresponded ICD-10 codes for drug use disorder (excluding pioid misuse, opioid dependence, and opioid poisoning, tobacco use disorder, and alcohol use disorder) are listed at https://www2.ccwdata.org/web/guest/condition-categories-other. |  |
| **Clinical conditions** |  |  |
| Mental disorder | Anxiety disorders (CCS 651), mood disorders (CCS 657), schizophrenia and other psychotic disorders (CCS 659) | HCUP CCS for *ICD-9-CM or ICD-10-CM* |
| Diabetes | Diabetes mellitus without complication (CCS 49), diabetes mellitus with complications (CCS 50) |  |
| Cardiovascular diseases | Heart valve disorders (CCS 96), coronary atherosclerosis and other heart disease (CCS 101), pulmonary heart disease (CCS 103), cardiac dysrhythmias (CCS 106), congestive heart failure; nonhypertensive (CCS 108), acute cerebrovascular disease (CCS 109), occlusion or stenosis of precerebral arteries (CCS 110), other and ill-defined cerebrovascular disease (CCS 111), peripheral and visceral atherosclerosis (CCS 114) |  |
| Hypertension | essential hypertension (CCS 98), hypertension with complications, and secondary hypertension (CCS 99) |  |
| Pulmonary condition | Pneumonia (except that caused by tuberculosis or sexually transmitted disease) (CCS 122), acute bronchitis (CCS 125), other upper respiratory infections (CCS 126), chronic obstructive pulmonary disease and bronchiectasis (CCS 127), asthma (CCS 128), pleurisy; pneumothorax; pulmonary collapse (CCS 130), respiratory failure; insufficiency; arrest (CCS 131), other lower respiratory disease (CCS 133) |  |
| Kidney disease | Nephritis; nephritis; renal sclerosis (CCS 156), acute and unspecified renal failure (CCS 157), chronic kidney disease (CCS 158), other diseases of kidney and ureters (CCS 161) |  |
| Gastrointestinal tract disorder | Gastrointestinal hemorrhage (CCS 153), other gastrointestinal disorders (CCS 155), digestive congenital anomalies (CCS 214) |  |
| Liver disease | Liver diseases (CCS 151) |  |
| Infections due to non-sterile opioid injection | 042, V08, 070.41, 070.44, 070.51, 070.54, 070.7x, V0262, 035, 040.0, 569.61, 681, 682, 785.4, 728.86, 038.2, 790.7, 421, 711.0, 730.0, 730.2, 320, A40.3, A46, A48.0, B17, B18.2, B19.2, B20, E08.52, E09.52, E10.52, E11.52, E13.52, G00, G01, G042, I33, I39, I70.36, I70.46, I70.56, I70.66, I70.76, I73.01, I96, K12.2, K94.02, K94.12, L02.01, L02.11, L02.21, L02.31, L02.41, L02.51, L02.61, L02.81, L02.91, L03, L98.3, M00, M72.6, M86, R78.81, Z21 | At least 1 inpatient, SNF, HHA, HOP, or carrier claim with disease code in any diagnostic position |
| Cognitive impairment | ICD-9 (ICD-10)  331.83 (G31.84) (mild cognitive impairment); 780.93 (R412, R413)(memory loss)  294.9 (F068) (cognitive disorder not otherwise specified); 437.7 (G45.4) (transient global amnesia); 290.x (F03.90, F05, F01.50, F01.51), 294.x (F04, F0280, F0281, F060, F068) (dementia) | At least 1 inpatient, SNF, HHA, HOP, or carrier claim with disease code in any diagnostic position |
| Frailty |  |  |

Abbreviations: E code, External Cause of Injury; *ICD-9-CM and ICD-10-CM*, *International Classification of Diseases, Ninth or Tenth Revision, Clinical Modification*; SNF, skilled nursing facility; HHA, home health agency; HOP, hospital outpatient; HCUP, Healthcare Cost and Utilization Project; CCS, Clinical Classification Software; DME, Duration Medical Equipment.
