# Supplementary material for: Association of injury after prescription opioid initiation with risk for opioid-related adverse events among older Medicare beneficiaries in the United States: A nested case-control study
Source: PLoS Med. 2022 Sep 22;19(9):e1004101. doi: 10.1371/journal.pmed.1004101 (PMC9498946; doi:10.1371/journal.pmed.1004101)
Supplement: S3 Table — (DOCX) [file pmed.1004101.s005.docx]

**S3 Table**. Association Between Incident Injury After Prescription Opioid Initiation and Subsequent Risk of Opioid Overdose and Risk of Opioid Use Disorder

| Exposure | Cases with OD  n=1547 (100%) | Matched Controls  n=6188 (100%) | Crude OR  (95% CI) | P value | Adjusted^a^ OR  (95% CI) | P value |
| --- | --- | --- | --- | --- | --- | --- |
| *Incident injury* |  |  |  |  |  |  |
| No | 656 (42.4) | 3628 (58.6) | Reference |  | Reference |  |
| Yes | 891 (54.0) | 2560 (41.4) | 2.31 (2.03-2.63) | <.001 | 1.67 (1.46-1.92) | <0.001 |
|  | Cases with OUD  n=2150 (100%) | Matched Controls  n=8598 (100%) | Crude OR  (95% CI) | P value | Adjusted^b^ OR  (95% CI) | P value |
| *Incident injury* |  |  |  |  |  |  |
| No | 1196 (55.6) | 5206 (60.6) | Reference |  | Reference |  |
| Yes | 954 (44.4) | 3392 (39.5) | 1.23 (1.15-1.42) | < .001 | 1.08 (0.95-1.22) | .23 |

Abbreviations: OR, odds ratio; OD, Opioid Overdose; OUD, opioid use disorder.

^a^Also adjusted for imbalanced covariates at follow-up, including diagnosis of chronic pain diagnosis, mental health disorders, cardiovascular disease, hypertension, pulmonary condition, kidney disease, gastrointestinal disorder, liver disease, respiratory infection, infection due to nonsterile opioid injection, cognitive impairment, frailty index, any hospital stay, any emergency room visit, any skilled nursing home visit, anticonvulsant use, anxiolytic use, as well as patterns of prescription opioid use (including use of chronic opioid use, use of high opioid dose, use of long-acting opioids, concurrent use of opioids and benzodiazepines).

^b^Also adjusted for imbalanced covariates at follow-up, including diagnosis of tobacco or alcohol use disorder, drug use disorder, chronic pain diagnosis, mental health disorders, gastrointestinal disorder, frailty index, emergency room visit, anticonvulsant use, as well as patterns of prescription opioid use (including use of chronic opioid use, use of high opioid dose, use of long-acting opioids, concurrent use of opioids and benzodiazepines)
