# Supplementary material for: Association of injury after prescription opioid initiation with risk for opioid-related adverse events among older Medicare beneficiaries in the United States: A nested case-control study
Source: PLoS Med. 2022 Sep 22;19(9):e1004101. doi: 10.1371/journal.pmed.1004101 (PMC9498946; doi:10.1371/journal.pmed.1004101)
Supplement: S4 Table — (DOCX) [file pmed.1004101.s006.docx]

**S4 Table**. Risk of Opioid Overdose by Recency of Injury in the Year Before the Index Date Among Older Patients With ≥1 Year of Follow-up.

| **Injury** | **Cases, No (%)**  n=888 | **Controls, No. (%)**  n=3552 | **Crude OR**  **(95% CI)** | ***P* value** | **Adjusted OR**^a^  **(95% CI)** | ***P* value** |
| --- | --- | --- | --- | --- | --- | --- |
| *Recency of Injury* |  |  |  |  |  |  |
| None | 413 (46.5) | 2279 (64.2) | Reference |  | Reference |  |
| Current (≤ 30 days) | 230 (25.9) | 220 (6.2) | 5.61 (4.52-6.96) | <.001 | 4.08 (3.22-5.17) | <.001 |
| New event | 110 (12.4) | 95 (2.7) | 6.21 (4.61-8.37) | <.001 | 5.39 (3.91-7.44) | <.001 |
| Recurrent event | 120 (13.5) | 125 (3.5) | 5.15 (3.92-6.78) | <.001 | 3.20 (2.37-4.33) | <.001 |
| Recent (31-90 days) | 65 (7.3) | 267 (7.5) | 1.36 (1.01-1.82) | .03 | 0.87 (0.63-1.20) | .40 |
| Past (91-180 days) | 79 (8.9) | 325 (9.2) | 1.30 (0.99-1.71) | .06 | 0.92 (0.69-1.23) | .57 |
| Remote (181-360 days) | 101 (11.4) | 461 (13.0) | 1.19 (0.93 -1.51) | .17 | 1.08 (0.73-1.59) | .36 |

Abbreviation: OR, odds ratio.

^a^Also adjusted for imbalanced covariates at follow-up, including diagnosis of chronic pain diagnosis, mental health disorders, cardiovascular disease, hypertension, pulmonary condition, kidney disease, gastrointestinal disorder, liver disease, respiratory infection, infection due to nonsterile opioid injection, cognitive impairment, frailty index, any hospital stay, any emergency room visit, any skilled nursing home visit, anticonvulsant use, anxiolytic use, as well as patterns of prescription opioid use (including use of chronic opioid use, use of high opioid dose, use of long-acting opioids, concurrent use of opioids and benzodiazepines).
