# Supplementary material for: Association of injury after prescription opioid initiation with risk for opioid-related adverse events among older Medicare beneficiaries in the United States: A nested case-control study
Source: PLoS Med. 2022 Sep 22;19(9):e1004101. doi: 10.1371/journal.pmed.1004101 (PMC9498946; doi:10.1371/journal.pmed.1004101)
Supplement: S5 Table — (DOCX) [file pmed.1004101.s007.docx]

**S5 Table**. Risk of Opioid Use Disorder by Recency of Injury in the Year Before the Index Date Among Older Adults With ≥1 Year of Follow-up.

| **Injury** | **Cases, No (%)**  n=1225 | **Controls, No. (%)**  n=8598 | **Crude OR**  **(95% CI)** | ***P* value** | **Adjusted OR**^a^  **(95% CI)** | ***P* value** |
| --- | --- | --- | --- | --- | --- | --- |
| *Recency of Injury* |  |  |  |  |  |  |
| None | 756 (61.7) | 4505 (65.3) | Reference |  | Reference |  |
| Current (≤ 30 days) | 105 (8.6) | 264 (5.4) | 1.72 (1.35-2.20) | <.001 | 1.32 (1.02-1.72) | 0.04 |
| New event | 34 (2.8) | 85 (1.7) | 1.74 (1.16-2.62) | <.001 | 1.52 (0.97-2.37) | 0.07 |
| Recurrent event | 71 (5.8) | 179 (3.7) | 1.72 (1.29-2.29) | <.001 | 1.24 (0.91-1.70) | .18 |
| Recent (31-90 days) | 98 (8.0) | 380 (7.8) | 1.11 (0.88-1.41) | .38 | 0.94 (0.73-1.21) | .61 |
| Past (91-180 days) | 112 (9.1) | 383 (7.8) | 1.25 (1.00-1.57) | .048 | 1.03 (0.81-1.31) | .83 |
| Remote (181-360 days) | 154 (12.6) | 609 (12.4) | 1.09 (0.90 -1.39) | .36 | 1.03 (0.83-1.26) | .81 |

Abbreviation: OR, odds ratio.

^a^Also adjusted for imbalanced covariates at follow-up, including diagnosis of tobacco or alcohol use disorder, drug use disorder, chronic pain diagnosis, mental health disorders, gastrointestinal disorder, frailty index, emergency room visit, anticonvulsant use, as well as patterns of prescription opioid use (including use of chronic opioid use, use of high opioid dose, use of long-acting opioids, concurrent use of opioids and benzodiazepines).
