# Supplementary material for: Association of injury after prescription opioid initiation with risk for opioid-related adverse events among older Medicare beneficiaries in the United States: A nested case-control study
Source: PLoS Med. 2022 Sep 22;19(9):e1004101. doi: 10.1371/journal.pmed.1004101 (PMC9498946; doi:10.1371/journal.pmed.1004101)
Supplement: S6 Table — (DOCX) [file pmed.1004101.s008.docx]

**S6 Table.** Characteristics of Patients with ORAEs During the Risk and Control Periods in a Case-Crossover Study Design

| **Characteristic** | Risk Period, No. (%)  n = 2436 | Control Period, No. (%)  n = 2436 | SDiff ^a^ |
| --- | --- | --- | --- |
| **Age group, y** |  |  | 0.000 |
| 65-74 | 1286 (52.8) | 1286 (52.8) |  |
| 75-84 | 728 (29.9) | 728 (29.9) |  |
| ≥85 | 421 (17.3) | 421 (17.3) |  |
| **Female** | 1669 (68.5) | 1669 (68.5) | 0.000 |
| **Race and ethnicity** |  |  | 0.000 |
| Black | 241 (9.9) | 241 (9.9) |  |
| White | 1951 (80.1) | 1951 (80.1) |  |
| Other^b^ | 246 (10.1) | 246 (10.1) |  |
| **Receiving low-income subsidy** | 731 (30.0) | 731 (30.0) | 0.000 |
| **US Region** |  |  | 0.000 |
| South | 1096 (45.0) | 1096 (45.0) |  |
| Northeast | 339 (13.9) | 339 (13.9) |  |
| Midwest | 514 (21.1) | 514 (21.1) |  |
| West | 487 (20.0) | 487 (20.0) |  |
| **Tobacco or alcohol use disorder** | 134 (5.5) | 83 (3.4) | 0.103 |
| **Drug use disorder** | 44 (1.8) | 12 (0.5) | 0.128 |
| **Chronic pain diagnosis** |  |  |  |
| Musculoskeletal | 1642 (67.4) | 1337 (54.9) | 0.260 |
| Neuropathic | 706 (29.0) | 577 (23.7) | 0.119 |
| Idiopathic | 543 (22.3) | 290 (11.9) | 0.280 |
| **Clinical condition** |  |  |  |
| Mental health disorders | 485 (19.9) | 363 (14.9) | 0.133 |
| Diabetes | 663 (27.2) | 577 (23.7) | 0.079 |
| Cardiovascular disease | 989 (40.6) | 682 (28.0) | 0.267 |
| Hypertension | 1223 (50.2) | 911 (37.4) | 0.259 |
| Pulmonary condition | 918 (37.7) | 604 (24.8) | 0.281 |
| Kidney disease | 453 (18.6) | 280 (11.5) | 0.200 |
| Gastrointestinal tract disorder | 448 (18.4) | 217 (8.9) | 0.280 |
| Liver disease | 114 (4.7) | 49 (2.0) | 0.148 |
| Respiratory infection | 448 (18.4) | 317 (13.0) | 0.148 |
| Infections due to nonsterile opioid injection | 141 (5.8) | 80 (3.3) | 0.117 |
| Cognitive impairment | 163 (6.7) | 97 (4.0) | 0.120 |
| **Polypharmacy** | 1055 (43.3) | 987 (40.5) | 0.057 |
| **Health care utilization** |  |  |  |
| Any hospital stay | 275 (11.3) | 112 (4.6) | 0.250 |
| Any ED visit | 353 (14.5) | 171 (7.0) | 0.248 |
| Any SNF stay | 100 (4.1) | 51 (2.1) | 0.118 |
| **Use of CNS medications** |  |  |  |
| Benzodiazepine | 336 (13.8) | 358 (14.7) | 0.026 |
| Non-benzodiazepine | 97 (4.0) | 168 (6.9) | 0.128 |
| Anticonvulsants | 424 (17.4) | 382 (15.7) | 0.045 |
| Antidepressants | 555 (22.8) | 504 (20.7) | 0.051 |
| Antipsychotics | 88 (3.6) | 71 (2.9) | 0.036 |
| Anxiolytics | 421 (17.3) | 460 (18.9) | 0.041 |
| **Prescription opioid use** |  |  |  |
| Opioid use | 1535 (63.0) | 923 (37.9) | 0.521 |
| High dose opioid (≥50 MME/daily) | 477 (19.6) | 382 (15.7) | 0.103 |
| Long-acting opioid | 188 (7.7) | 73 (3.0) | 0.210 |
| Opioid plus benzodiazepine | 312 (12.8) | 253 (10.4) | 0.076 |

Abbreviations: SDiff= standardized difference; ED, emergency department; SNF, skilled nursing facility; CNS, central nervous system; MME, morphine milligram equivalent.

^a^Covariates with SDiff >0.1 represent meaningful differences between case and control groups.

^b^Included Hispanic, Asian, Pacific Islander, and Native American individuals.
