# Supplementary material for: Association of injury after prescription opioid initiation with risk for opioid-related adverse events among older Medicare beneficiaries in the United States: A nested case-control study
Source: PLoS Med. 2022 Sep 22;19(9):e1004101. doi: 10.1371/journal.pmed.1004101 (PMC9498946; doi:10.1371/journal.pmed.1004101)
Supplement: S7 Table — (DOCX) [file pmed.1004101.s009.docx]

**S7 Table.** Findings of Case-Crossover Analyses of the Association Between Injury and Risk of Opioid-Related Adverse Event

| **Exposure** | **Risk Period**  **No. (%)**  n=2346 | **Control Period**  **No. (%)**  n=2346 | **Crude OR**  **(95% CI)** | **P-value** | **Adjusted^a^ OR**  **(95% CI)** | **P-value** |
| --- | --- | --- | --- | --- | --- | --- |
| *Incident Injury* |  |  |  |  |  |  |
| No | 1940 (82.7) | 2169 (92.5) | Reference |  | Reference |  |
| Yes | 406 (17.3) | 177 (7.5) | 2.91 (2.36-3.58) | <0.001 | 2.31 (1.79-2.97) | <0.001 |

Abbreviation: OR, odds ratio.

^a^ Also adjusted for imbalanced covariates (shown in eTable7), including tobacco or alcohol use disorder, drug use disorder, type of chronic pain diagnosis, mental health disorders, cardiovascular disease, hypertension, pulmonary condition, kidney disease, gastrointestinal tract disorder, liver disease, respiratory infection, infection due to nonsterile opioid injection, cognitive impairment, hospital stay, hospital stay, emergency department visit, skilled nursing facility, use of non-benzodiazepine, and patterns of prescription opioid use (including use of high opioid dose and use of long-acting opioids).
